# Supplementary material for: Management of Pyrenophora teres f. teres, the Causal Agent of Net Form Net Blotch of Barley, in A Two-Year Field Experiment in Central Italy
Source: Pathogens. 2022 Feb 24;11(3):291. doi: 10.3390/pathogens11030291 (PMC8954409; doi:10.3390/pathogens11030291)
Supplement: Supplementary file 1 [file pathogens-11-00291-s001.zip › pathogens-1596164-supplementary.pdf]

Management of *Pyrenophora teres* f. *teres*, the causal agent of net form net blotch of barley, in a two-year field experiment in central Italy

Francesco Tini, Lorenzo Covarelli, Giacomo Ricci, Emilio Balducci, Maurizio Orfei, Giovanni Beccari

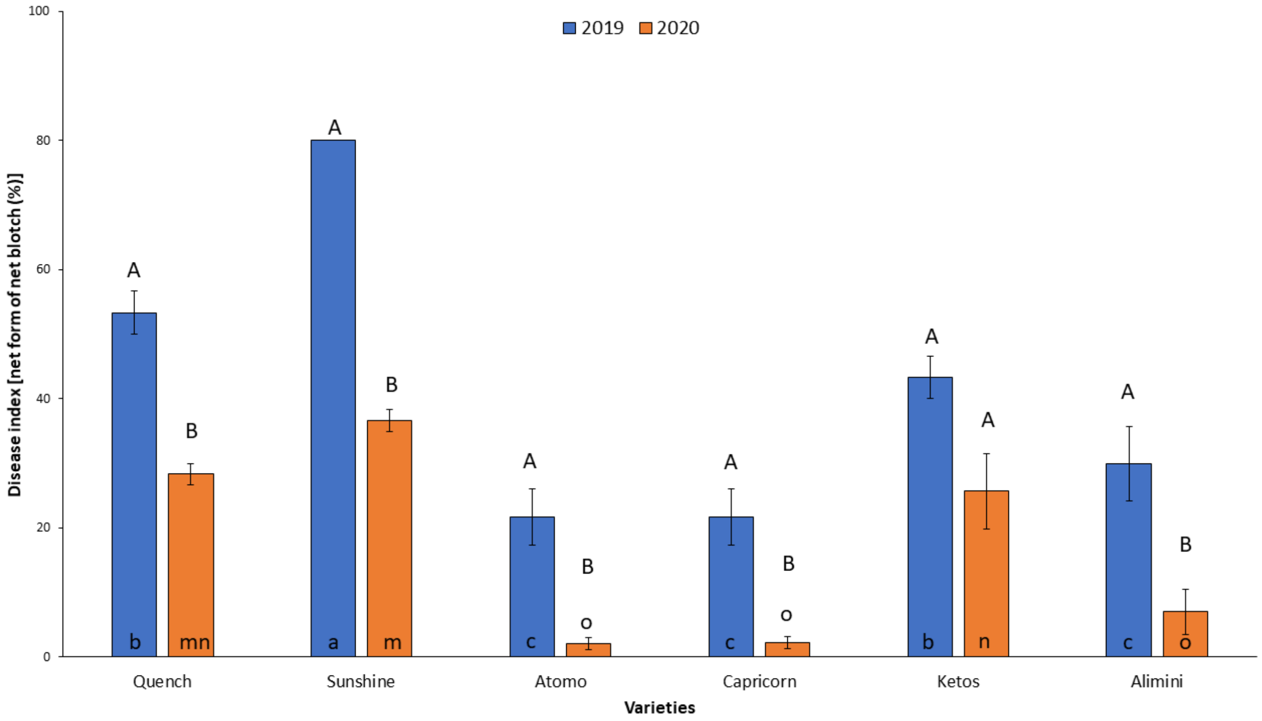

Figure S1. Average (three replicates) of disease index [net form net blotch symptoms (%)] observed during 2019 and 2020 on the untreated controls of the six barley varieties. Within the same year (a – c; m – o) or between the years (A – B), means with the same letters are not significantly different at $p \leqslant 0.05$ based on Duncan's multiple comparison test.

Table S1. Primers used in PCR assays and qPCR analysis.

Primer	Sequence (5'-3')	Size	Annealing T(°C)	Used in this study	References
ITS1	TCCGTAGGTGAACCTGCGG	290 bp	56	PCR	White et al., 1990
ITS4	TCCTCCGCTTATTGATATGC				
PttQ4-F	CGTCCCGCCGAAATTTTGTA	173 bp	60	PCR/qPCR	Poudel et al., 2017
PttQ4-R	CAAGGACTTACGCGCTCAAA				
PtmQ10-F	TGCTGTGGACTTAGACGAGG	260 bp	62	PCR	Pudel et al., 2017
PtmQ10-R	TGGGGATCCTTGACCAACTC				
Hor1-F	TCTCTGGGTTTGAGGGTGAC		61	qPCR	Nicolaisen et al., 2009
Hor2-R	GGCCCTTGTACCAGTCAAGGT				
